# Application of the neonatal ‘floppy module’ to older infants: can it be used for differential diagnosis?

**DOI:** 10.1007/s00431-025-06483-0

**Published:** 2025-10-07

**Authors:** Costanza Cutrona, Martina Sbarbati, Martina Malaspina, Chiara Velli, Francesca Sini, Roberta Onesimo, Giorgia Coratti, Domenico M. Romeo, Marika Pane, Eugenio Mercuri

**Affiliations:** 1 Pediatric Neurology, Università Cattolica del Sacro Cuore, Rome, Italy; 2 Nemo Clinical Center, Fondazione Policlinico Universitario Agostino Gemelli IRCCS, Rome, Italy; 3 Pediatric Neurology and Psychiatry Unit, Fondazione Policlinico Universitario Agostino Gemelli IRCCS, Rome, Italy; 4 Department of Pediatric Neurorehabilitation, Fondazione Santa Lucia IRCCS, Rome, Italy; 5 Pediatric Unit, Fondazione Policlinico Universitario Agostino Gemelli IRCCS, Rome, Italy; 6 Center for Rare Disease, Fondazione Policlinico Universitario Agostino Gemelli IRCCS, Rome, Italy

**Keywords:** Hypotonia, Floppy infant module, Neuromuscular disorders, Neonatal neurological examination

## Abstract

In this study we applied the ‘floppy module’, originally developed for newborns with hypotonia, to older infants in order to establish the range of findings in both low-risk infants and in those with hypotonia due to different etiologies. Data were collected from 413 assessments obtained in 159 low-risk infants assessed at different ages between the age of 3 and 24 months. The distribution of findings in the low-risk infants was similar to the data previously obtained in low-risk newborns, with all assessments having optimal findings (column 3) on all items. The only item with a small number of findings outside column 3 was tendon reflexes. The module was also applied to a cohort of 57 infants with hypotonia older than 3 months in order to identify individual items that may be useful in differential diagnosis. Items assessing antigravity movements were the most sensitive to identify neuromuscular disorders. Dysmorphic features and other organ involvement were most suggestive of genetic disorder, while seizures were a reliable indicator of CNS involvement.

*Conclusion*: Our findings suggest that the neonatal floppy module can be reliably administered also in infants from the age of 3 months. The pilot application of the module in infants with hypotonia from the age of 3 months also suggested that the module could be used to help the clinician in the differential diagnosis of infants with hypotonia.

**Table:** 

**What is Known:** • *Hypotonia is a relatively common finding in the neonatal period and can be due to multiple causes.* • *As hypotonia can be observed also after the neonatal period and clinicians often face the challenge of differential diagnosis, the question has arisen whether the ‘floppy module’ validated in newborns could also be used in infants.*
**What is New:** • *Most of the infants with hypotonia, between the age of 3 to 24 months, have a diagnosis of neuromuscular disorders.* • *Most of the infants with CNS involvement and with genetic diagnosis, who may have shown hypotonia in the neonatal period, by 6 to 12 months have normal or increased tone.*

**What is Known:**

• *Hypotonia is a relatively common finding in the neonatal period and can be due to multiple causes.*

• *As hypotonia can be observed also after the neonatal period and clinicians often face the challenge of differential diagnosis, the question has arisen whether the ‘floppy module’ validated in newborns could also be used in infants.*

**What is New:**

• *Most of the infants with hypotonia, between the age of 3 to 24 months, have a diagnosis of neuromuscular disorders.*

• *Most of the infants with CNS involvement and with genetic diagnosis, who may have shown hypotonia in the neonatal period, by 6 to 12 months have normal or increased tone.*

## Introduction

Hypotonia is a relatively common finding in the neonatal period and can be due to multiple causes, including not only peripheral involvement of muscle, nerve, or motoneurons but also syndromic and metabolic diseases [1–5]. In a number of cases, it can also be found in newborns with central nervous system involvement, including some who will later develop spasticity [6–8]. Several studies have emphasized that a thorough clinical examination is essential for differential diagnosis and can aid in distinguishing features of central versus peripheral nervous system involvement [1–5].

In a previous study, we proposed a new module specifically designed to be used in the neonatal period in newborns with hypotonia, reporting its application to a cohort of 143 low-risk newborns and in 24 floppy newborns with different conditions (neuromuscular disorder, genetic conditions, hypoxic ischemic encephalopathy, CNS malformations and metabolic disease) [9]. The module was developed to be easily used in a clinical setting as an add-on module to the main Hammersmith neonatal neurological examination (HNNE), providing extra items to identify signs suggestive of neuromuscular conditions [9]. The neonatal module has been increasingly used in neonatal units and has also been applied to newborns with spinal muscular atrophy identified by neonatal screening [10, 11].

As hypotonia can be observed also after the neonatal period and clinicians often face the challenge of differential diagnosis, the question has arisen whether the ‘floppy module’ validated in newborns could also be used after the neonatal period.

The aim of this study was to apply the same module in low-risk infants in order to establish the reproducibility of the scoring system after the neonatal period. The module was also administered to a cohort of infants presenting with hypotonia at or after 3 months of age to assess whether the included items could help identify potential differences among patients with varying diagnoses.

## Materials and methods

The present study was part of a larger follow-up research project carried out at our unit aimed to validate neonatal and infant neurological examinations. The study protocol was approved by the ethics committee of our institution (Fondazione Policlinico Gemelli) (ID 0036454/22), and informed written consent was obtained from the parents in all cases.

### Floppy module

The main section of the module includes 11 items. Nine of the 11 assess antigravity movements and contractures (in both upper and lower limbs), tendon reflexes, facial muscles, respiratory pattern, and sucking and swallowing. This section also includes two items on possible antenatal findings (fetal movements and amniotic fluid).

The 11 items can be scored reporting the findings in each item on one of the three columns in the form. These were based on the distribution of findings in a cohort of low-risk infants: the first column includes findings considered suboptimal, i.e. those who were found in less than 5% of the low-risk newborns, the second column is for intermediate findings (found in more than 5% but less than 15%), and the third column includes the findings that are considered optimal. The add-on module can be easily administered within 3 min.

The module also includes a separate section to focus on other aspects, captured by the main neurological examination, that could contribute to identify specific signs for differential diagnosis.

### Application of the neonatal module to older low-risk infants

The scoring system of the form was based on the distribution of findings originally obtained in low-risk newborns; in order to confirm that a similar distribution was also applicable to infants beyond the neonatal period, we applied the module to a cohort of low-risk term-born infants used as controls in a parallel study, with no antenatal or perinatal risk factors, a normal neurological examination and a typical neurodevelopmental outcome at 24 months. As infants born prematurely tend to have some degree of hypotonia in the neonatal period that may persist beyond the neonatal period, we also applied the tool to a separate cohort of low-risk preterm infants, therefore excluding those with abnormal findings at the cranial ultrasound scans, such as grade II–IV hemorrhages or moderate/severe white matter lesions.

Clinical and demographic information was routinely collected.

### Pilot application to floppy infants after the neonatal period

The ‘floppy module’ was subsequently applied to a cohort of infants who had signs of generalized hypotonia on the main neurological exam (HINE), irrespective of their diagnosis, followed in our outpatient clinics following infants and children with neuromuscular disorders, cerebral palsy (CP), and genetic disorders.

### Statistical analysis

Demographic and clinical features were summarized using frequencies and percentages for categorical variables and means with standard deviations for continuous variables. The diagnostic performance of the module for detecting neuromuscular disorders was evaluated through the calculation of sensitivity and specificity metrics. Sensitivity was defined as the proportion of true positive cases (patients with confirmed neuromuscular disorders correctly identified by the module) among all patients with actual neuromuscular disorders, representing the module’s ability to correctly identify disease presence. Specificity was calculated as the proportion of true negative cases (patients without neuromuscular disorders correctly classified as negative by the module) among all patients without the condition, indicating the module’s capacity to correctly exclude disease absence.

## Results

### Application to low-risk infants: term born infants

Data were collected from 192 longitudinal assessments, obtained by a total of 48 low-risk infants (F = 20; M = 28) with no comorbidities reported, assessed at different time points (3, 6, 9 and 12 months). Gestational age ranged from 37 to 41 weeks (mean 38.20 ± 1.28). Birth weight for GA, *n* (%): 47 (98.92%) AGA, 1 (2.08%) SGA. All low-risk term infants had findings in column 3 in all items, except for the item assessing tendon reflexes, with three assessments (2%) showing decreased reflexes (column 2).

### Application to low-risk infants: prematurely born infants

Data were collected from 221 assessments, obtained by a total of 111 low-risk preterm infants (F = 63; M = 75). Of the total cohort, 38 have been clinically evaluated once, 43 were evaluated longitudinally twice, 23 were seen longitudinally three times, and seven were seen at four different time points. The demographic characteristics of the sample are reported in Table 1.

**Table 1:** Characteristics of the low-risk preterm cohort in the whole cohort (GA = gestational age, AGA = appropriate for gestational age, SGA = small for gestational age)

	Low risk preterm cohort (*n *= 111)	
*Gender n (%)*	Male	48 (43%)
	Female	63 (57%)
*Gestational age n (%)*	Extremely preterm (< 28 wk)	28 (25%)
	Very preterm (28–31 wk)	75 (68%)
	Moderate preterm (32–33 wk)	5 (5%)
	Late preterm (32–36 wk)	3 (3%)
*Birth weight for GA n (%)*	AGA	82 (77%)
	SGA	24 (23%)
*5’ APGAR for GA Mean (SD)*	Extremely preterm (< 28 wk)	7,84 (1,16)
	Very preterm (28–31 wk)	8,34 (1,21)
	Moderate preterm (32–33 wk)	8,25 (0,95)
	Late preterm (32–36 wk)	9 (0)

All prematurely born infants had findings in column 3 in all items, irrespective of the gestational age at birth, with the exception of the item assessing tendon reflexes. In 32 assessments (15%), reflexes were reported as mildly brisk and easily elicitable. Table 1 shows details of the characteristics of the population.

### Pilot application in hypotonic infants

Hypotonia at the age of 3 months or later was found in 57 patients, 43 with a diagnosis of NMD, seven of genetic syndromes (Down syndrome and Prader-Willi syndrome) and seven had cerebral palsy (CP).

The neuromuscular cohort included 43 patients who were assessed at several times, for a total of 150 assessments within the second year of life. These included infants affected by SMA I (72%), congenital myopathies (14%), SMARD1 (7%), and myotonic dystrophy (5%). In all the assessments, there was persistence of hypotonia, irrespective of the age.

The CP group included seven patients. The assessments showing persistent hypotonia were all performed < 12 months; in the follow-up assessments, the tone was gradually increasing in all CP patients.

The syndromic cohort included seven patients for a total of 19 assessments.

Table 2 gives the frequency distribution of findings on the floppy infant module in the clinical subgroups, according to the final diagnosis.

**Table 2:** Frequency distribution of findings on the floppy infant module in the clinical subgroups, according to the final diagnosis

		Neuromuscular	Cerebral palsy	Genetic
		150 assessments/43 patients	7 assessments/7 patients	19 assessments/7 patients
*Antigravity movements (lower limbs)*	*Normal*	32 (21%)	7 (100%)	19 (100%)
	*Abnormal*	118 (79%)	0 (0%)	0 (0%)
*Antigravity movements (upper limbs)*	*Normal*	84 (56%)	7 (100%)	19 (100%)
	*Abnormal*	69 (44%)	0 (0%)	0 (0%)
*Tendon reflexes*	*Normal*	0 (0%)	7 (100%)	13 (68%)
	*Abnormal*	150 (100%)	0 (0%)	6 (32%)
*Contractures lower limbs*	*Normal*	98 (65%)	7 (100%)	19 (100%)
	*Abnormal*	52 (34%)	0 (0%)	0 (0%)
*Contractures upper limbs*	*Normal*	122 (81%)	7 (100%)	19 (100%)
	*Abnormal*	28 (19%)	0 (0%)	0 (0%)
*Facial muscles*	*Normal*	87 (58%)	7 (100%)	7 (37%)
	*Abnormal*	63 (32%)	0 (0%)	12 (63%)
*Respiratory pattern*	*Normal*	11 (7%)	7 (100%)	18 (95%)
	*Abnormal*	139 (93%)	0 (0%)	1 (5%)
*Sucking*	*Normal*	66 (44%)	4 (57%)	15 (79%)
	*Abnormal*	84 (56%)	0 (0%)	4 (21%)
*Swallowing*	*Normal*	71 (47%)	6 (86%)	17 (90%)
	*Abnormal*	79 (53%)	1 (14%)	2 (10%)
*Reduced fetal movements*	*Normal*	118 (79%)	7 (100%)	19 (100%)
	*Abnormal*	32 (21%)	0 (0%)	0 (0%)
*Amniotic fluid*	*Normal*	132 (88%)	7 (100%)	19 (100%)
	*Abnormal*	18 (12%)	0 (0%)	0 (0%)
*Seizures*	*Normal*	150 (100%)	2 (29%)	19 (100%)
	*Abnormal*	0 (0%)	5 (71%)	0 (0%)
*Dysmorphism*	*Normal*	150 (100%)	7 (100%)	0 (0%)
	*Abnormal*	0 (0%)	0 (0%)	19 (100%)
*Other organ involvement*	*Normal*	150 (100%)	7 (100%)	0 (0%)
	*Abnormal*	0 (0%)	0 (0%)	19 (100%)

Infants with neuromuscular disorders frequently demonstrated multiple abnormal findings, particularly in items evaluating weakness, contractures, and antenatal history. Among those with abnormal findings who underwent repeated evaluations, results were consistently concordant across different ages.

The diagnostic performance metrics demonstrate substantial variability across clinical features and diagnostic categories. For NMD, sensitivity values ranged from 0.0 to 1.0, with respiratory pattern achieving the highest combined performance (sensitivity 0.92, specificity 0.96). Five features exhibited perfect specificity (1.0): antigravitary movements, contractures, reduced fetal movements, amniotic fluid abnormalities, and dysmorphism, while their corresponding sensitivities varied dramatically from 0.0 to 0.81. Tendon reflexes demonstrated perfect sensitivity (1.0) paired with moderate specificity (0.76). Three features (seizures, dysmorphism, other organ involvement) showed zero sensitivity for NMD detection.

For genetic conditions, performance metrics exhibited a bimodal distribution. Dysmorphism and other organ involvement achieved perfect performance across both sensitivity and specificity (1.0), while facial muscles demonstrated moderate values (sensitivity 0.63, specificity 0.59). The remaining features displayed sensitivity values ranging from 0.0 to 0.31, with specificity values spanning 0.04 to 0.96. Notably, six features registered zero sensitivity for genetic condition detection.

CP detection showed predominantly low sensitivity values, with ten of twelve features demonstrating zero sensitivity. Seizures provided the highest sensitivity (0.71) with perfect specificity (1.0), while sucking achieved moderate sensitivity (0.42) with specificity of 0.47. Specificity values for CP detection ranged from 0.07 to 1.0, with four features achieving values above 0.80.

Table 3 shows the sensitivity and specificity of the individual findings.

**Table 3:** Sensitivity and specificity of the individual findings

	Neuromuscolar		Cerebral palsy		Genetic	
	Sensitivity	Specificity	Sensitivity	Specificity	Sensitivity	Specificity
*Antigravitary upper/lower movements*	0.81	1	0	0.22	0	0.27
*Tendon reflexes*	1	0.76	0.31	0.04	0	0.07
*Contractures upper/lower limbs*	0.37	1	0	0.64	0	0.69
*Facial muscles*	0.42	0.53	0.63	0.59	0	0.55
*Respiratory pattern*	0.92	0.96	0.05	0.11	0	0.17
*Sucking*	0.56	0.73	0.21	0.44	0.42	0.47
*Swallowing*	0.52	0.88	0.10	0.49	0.14	0.52
*Reduced fetal movements*	0.21	1	0	0.79	0	0.81
*Amniotic fluid*	0.12	1	0	0.88	0	0.89
*Seizures*	0	0.19	0	0.96	0.71	1
*Dysmorfism*	0	0.26	1	1	0	0.88
*Other organ involvement*	0	0.26	1	1	0	0.88

## Discussion

The HNNE and the infant version (HINE), to be used after the neonatal period, were developed as structured scorable examinations that include several aspects of neurological function (tone, behavior, movements etc.). The combined sequential use of HNNE and HINE allows one to follow the evolution of neurological signs and has mostly been used worldwide in the follow-up of both low-risk preterm and full-term infants with brain lesions [12]. As hypotonia is common in the neonatal period, the ‘floppy module’ was developed to complement the HNNE with additional items. The new items provide information on aspects of neurological signs that are more relevant to newborns with neuromuscular disorders or other diagnoses such as genetic disorders [9]. The question has arisen of whether the ‘floppy module’, originally developed and validated for the neonatal period only, could also be used in conjunction with the Hammersmith infant neurological examination (HINE) [13, 14], for the differential diagnosis after the neonatal period.

Our study shows that the floppy module, initially developed for neonates, can be reliably applied also to older infants. Its application to a cohort of low-risk infants born at term age assessed at the age of 3 months or after showed that, similarly to what was observed in low-risk newborns [9], all the items had always findings in the 3rd column (optimal), irrespective of the age at assessment. Only 2% scored in the 2nd column (suboptimal) in one item, with none scoring in the first column in any item. The only item in which there were findings in the 2nd column was ‘reflexes’ as in a small proportion of infants these could be ‘felt’ but not seen as observed in the validation.

As there is evidence that infants born prematurely have a different pattern of findings in the items assessing tone and other neurological aspects compared to term-born infants [15, 16], we also investigated possible differences in the items included in the floppy module. The scores obtained in the infants born prematurely, however, did not show obvious differences with those obtained in term infants. This held true in the whole cohort, irrespective of their gestational age at birth or post-natal age at assessment. The only difference was related to the item assessing reflexes, as none of the prematurely born infants had weak reflexes and 15% had minimally brisk reflexes as often observed in prematurely born infants [17–19].

These results therefore suggest that, as reported for the neonatal module, signs of contractures, weakness, or other abnormal signs assessed by the module do not generally occur in a low-risk cohort, irrespective of their gestational age at birth. Their presence, especially if abnormal scores are found in more than one item, should therefore always raise concern.

The application of the module to infants who were found to have generalized hypotonia on the HINE provided additional information that could be used for differential diagnosis. It is not surprising that, between the age of 3 and 24 months, most of the infants with hypotonia had a diagnosis of neuromuscular disorders. These numbers partly reflect the fact that most of the infants with CNS involvement, who may have shown hypotonia in the neonatal period, by 6 to 12 months have normal or increased tone. The same also applies to some genetic diagnoses that are also associated with gradual improvement of the neonatal hypotonia.

Even with these limitations, the application of the floppy module helped to highlight which signs are more relevant in the differential diagnosis. We confirmed that antigravity movements, followed by contractures, were the most sensitive signs to identify patients with NMD and these were not age specific. The specificity of antigravity movements to detect a neuromuscular disorder was 1 with also a high sensitivity, indicating that while absent antigravity movements were always specifically associated with a NMD (true positive rate of 100% among those with absent movements), their presence cannot always exclude a NMD (meaning some patients with NMD still retained antigravity movements, resulting in false negatives). This was generally occurring in infants with congenital myopathies and milder myotonic dystrophies. The assessment of antigravity movements in the floppy module is different from those assessing quantity and quality in the main HINE examination. It is only the detection of absent/severely reduced antigravity movements that indicate weakness and therefore neuromuscular disorders. In contrast, reduced movements, such as poor repertoire or other abnormal patterns, if associated with even isolated antigravity movements, are more suggestive of other causes, such as brain lesions.

Not surprisingly, the presence of dysmorphisms and other organ involvement was most suggestive of genetic disorder [20]. These features demonstrated perfect sensitivity and specificity (1.0 each), meaning they correctly identified all genetic disorder cases when present (no false negatives) and were never found in non-genetic cases (no false positives). Other signs, such as sucking and swallowing abnormalities, were common across different diagnoses (showing low specificity due to high false positive rates across multiple conditions).

The presence of seizures was a reliable indicator of CNS involvement as was mainly found in infants who developed CP. Seizures showed high specificity for CP detection (1.0), meaning no infants without CP exhibited seizures (zero false positives), though the moderate sensitivity (0.71) indicates that approximately 29% of CP cases did not present with seizures (false negatives). The number of infants affected by CP who showed hypotonia later in the first months of age was relatively small as the great majority of the infants who develop CP and have neonatal hypotonia generally show a gradual increase in tone over the first months after birth. This held true in our cohort as the assessments showing hypotonia were all performed before the age of 12 months and, in the same infants, the subsequent examinations showed a gradual increase in tone [21]. Interestingly, all these patients had mild to moderate white matter lesions and ventricular dilatation and none had the more severe white matter changes or basal ganglia lesions that are known to be associated with an earlier onset of hypertonia [22, 23].

In conclusion, our new findings suggest that the neonatal floppy module can be reliably administered also in infants between 3 and 24 months, as the distribution of findings in the low-risk older cohort was similar to the neonatal one, irrespective of the age at assessment or of the gestational age at birth. The pilot application of the module in infants with hypotonia at the age of 3 months or later also suggested that the module could be used to help the clinician to perform an accurate differential diagnosis in case of hypotonia. Implementation of this tool in clinical practice could also be useful in assessing response to therapy, especially in floppy infants with genetic syndromes such as spinal muscular atrophy for which therapies are now available. More structured studies using new genetic tools in larger cohorts could help to better characterize the spectrum of clinical phenotypes and etiologies associated with hypotonia [24–26].

## Data Availability

The data that support the findings of this study are available upon reasonable request to the corresponding author.

## Abbreviations

CNS: Central nervous system
CP: Cerebral palsy
HINE: Hammersmith infant neurological examination
HNNE: Hammersmith neonatal neurological examination
NMD: Neuro-muscolar disorders
SMA: Spinal muscolar atrophy
SMARD1: Spinal muscular atrophy with respiratory distress type 1

## References

[1] Vasta I, Kinali M, Messina S, Guzzetta A, Kapellou O, Manzur A, Cowan F, Muntoni F, Mercuri E (2005) Can clinical signs identify newborns with neuromuscular disorders? J Pediatr 146(1):73–79. 10.1016/j.jpeds.2004.08.047 15644826 10.1016/j.jpeds.2004.08.047

[2] Bodensteiner JB (2008) The evaluation of the hypotonic infant. Semin Pediatr Neurol 15(1):10–20. 10.1016/j.spen.2008.01.003 18342256 10.1016/j.spen.2008.01.003

[3] Laugel V, Cossée M, Matis J, de Saint-Martin A, Echaniz-Laguna A, Mandel JL, Astruc D, Fischbach M, Messer J (2008) Diagnostic approach to neonatal hypotonia: retrospective study on 144 neonates. Eur J Pediatr 167(5):517–523. 10.1007/s00431-007-0539-3 17641914 10.1007/s00431-007-0539-3

[4] Lisi EC, Cohn RD (2011) Genetic evaluation of the pediatric patient with hypotonia: perspective from a hypotonia specialty clinic and review of the literature. Dev Med Child Neurol 53(7):586–599. 10.1111/j.1469-8749.2011.03918.x 21418198 10.1111/j.1469-8749.2011.03918.x

[5] Dubowitz V (1968) The floppy infant–a practical approach to classification. Dev Med Child Neurol 10(6):706–710. 10.1111/j.1469-8749.1968.tb02967.x 5715647 10.1111/j.1469-8749.1968.tb02967.x

[6] Ricci D et al (2006) Neurological examination at 6 to 9 months in infants with cystic periventricular leukomalacia. Neuropediatrics 37(4):247–252. 10.1055/s-2006-924581 17177152 10.1055/s-2006-924581

[7] Mercuri E et al (2004) Neonatal cerebral infarction and neuromotor outcome at school age. Pediatrics 113(1):95–100. 10.1542/peds.113.1.95 14702455 10.1542/peds.113.1.95

[8] Haataja L, Mercuri E, Guzzetta A, Rutherford M, Counsell S, Frisone MF, Cioni G, Cowan F, Dubowitz L (2001) Neurologic examination in infants with hypoxic-ischemic encephalopathy at age 9 to 14 months: use of optimality scores and correlation with magnetic resonance imaging findings. J Pediatr 138(3):332–337. 10.1067/mpd.2001.111325 11241038 10.1067/mpd.2001.111325

[9] Cutrona C et al (2022) Assessing floppy infants: a new module. Eur J Pediatr 181(7):2771–2778. 10.1007/s00431-022-04476-x 35504981 10.1007/s00431-022-04476-x PMC9192404

[10] Pane M et al (2022) Neurological assessment of newborns with spinal muscular atrophy identified through neonatal screening. Eur J Pediatr 181(7):2821–2829. 10.1007/s00431-022-04470-3 35522315 10.1007/s00431-022-04470-3 PMC9192449

[11] Pane M et al (2024) Early neurological signs in infants identified through neonatal screening for SMA: do they predict outcome? Eur J Pediatr 183(7):2995–2999. 10.1007/s00431-024-05546-y 38634892 10.1007/s00431-024-05546-y PMC11192803

[12] Dubowitz L, Mercuri E, Dubowitz V (1998) An optimality score for the neurologic examination of the term newborn. J Pediatr 133(3):406–416. 10.1016/s0022-3476(98)70279-3 9738726 10.1016/s0022-3476(98)70279-3

[13] Haataja L, Mercuri E, Regev R, Cowan F, Rutherford M, Dubowitz V, Dubowitz L (1999) Optimality score for the neurologic examination of the infant at 12 and 18 months of age. J Pediatr 135(2):153–161. 10.1016/s0022-3476(99)70016-8 10431108 10.1016/s0022-3476(99)70016-8

[14] Haataja L, Cowan F, Mercuri E, Bassi L, Guzzetta A, Dubowitz L (2003) Application of a scorable neurologic examination in healthy term infants aged 3 to 8 months. J Pediatr 143(4):546. 10.1067/S0022-3476(03)00393-7 14603891 10.1067/S0022-3476(03)00393-7

[15] Romeo DMM et al (2007) Application of a scorable neurological examination to near-term infants: longitudinal data. Neuropediatrics 38(5):233–238. 10.1055/s-2007-1004520 18330837 10.1055/s-2007-1004520

[16] Romeo DM et al (2012) Neurologic assessment tool for screening preterm infants at term age. J Pediatr 161(6):1166–1168. 10.1016/j.jpeds.2012.07.037 22910101 10.1016/j.jpeds.2012.07.037

[17] Romeo DM et al (2022) Hammersmith infant neurological examination in low-risk infants born very preterm: a longitudinal prospective study. Dev Med Child Neurol 64(7):863–870. 10.1111/dmcn.15201 35298030 10.1111/dmcn.15201

[18] Kelly CE et al (2019) Brain structure and neurological and behavioural functioning in infants born preterm. Dev Med Child Neurol 61(7):820–831. 10.1111/dmcn.14084 30536389 10.1111/dmcn.14084

[19] Forslund M, Bjerre I (1983) Neurological assessment of preterm infants at term conceptional age in comparison with normal full-term infants. Early Hum Dev 8(3–4):195–208. 10.1016/0378-3782(83)90002-6 6641565 10.1016/0378-3782(83)90002-6

[20] DiMario FJ (1989) Genetic diseases in the etiology of the floppy infant. Rhode Island Med J 72(10):357–359 2595190

[21] Cooper MS, Antolovich GC, Fahey MC, Amor DJ (2024) Hypotonic cerebral palsy. Child Care Health Dev 50(3):e13258. 10.1111/cch.13258 38558298 10.1111/cch.13258

[22] Hidalgo Robles Á, Paleg GS, Livingstone RW (2024) Identifying and evaluating young children with developmental central hypotonia: an overview of systematic reviews and tools. Healthcare. 10.3390/healthcare12040493 38391868 10.3390/healthcare12040493 PMC10887882

[23] Straathof EJM, Hamer EG, Hensens KJ, La Bastide-van Gemert S, Heineman KR, Hadders-Algra M (2022) Development of muscle tone impairments in high-risk infants: associations with cerebral palsy and cystic periventricular leukomalacia. Eur J Paediatr Neurol 37:12–18. 10.1016/j.ejpn.2021.12.015 35007848 10.1016/j.ejpn.2021.12.015

[24] Prasad AN, Prasad C (2011) Genetic evaluation of the floppy infant. Semin Fetal Neonatal Med 16(2):99–108. 10.1016/j.siny.2010.11.002 21131247 10.1016/j.siny.2010.11.002

[25] Sharma S, Repnikova E, Noel-MacDonnell JR, LePichon J-B (2021) Diagnostic yield of genetic testing in 324 infants with hypotonia. Clin Genet 100(6):752–757. 10.1111/cge.14057 34480364 10.1111/cge.14057 PMC9291145

[26] Morton SU et al (2022) Multicenter consensus approach to evaluation of neonatal hypotonia in the genomic era: a review. JAMA Neurol 79(4):405–413. 10.1001/jamaneurol.2022.0067 35254387 10.1001/jamaneurol.2022.0067 PMC10134401

